# Mediating role of sensory differences in the relationship between autistic traits and internalizing problems

**DOI:** 10.1186/s40359-022-00854-0

**Published:** 2022-06-13

**Authors:** Yurika Tsuji, Satoko Matsumoto, Aya Saito, Shu Imaizumi, Yoko Yamazaki, Tetsuyuki Kobayashi, Yoko Fujiwara, Mika Omori, Masumi Sugawara

**Affiliations:** 1 Graduate School of Humanities and Sciences, Ochanomizu University, 2-1-1 Otsuka, Bunkyo, Tokyo, Japan; 2 Institute for Education and Human Development, Ochanomizu University, Bunkyo, Tokyo, Japan; 3 Faculty of Core Research, Ochanomizu University, Bunkyo, Tokyo, Japan; 4 Institute for Human Life Innovation, Ochanomizu University, Bunkyo, Tokyo, Japan; 5 Graduate School of Arts and Letters, Tohoku University, Sendai, Miyagi Japan; 6 Faculty of Human Studies, Shirayuri University, Chofu, Tokyo, Japan

**Keywords:** Autism spectrum disorder, Sensory differences, Sensory profile, Suffering, Internalizing problems

## Abstract

**Background:**

Sensory differences are related to the autistic traits, and previous studies have shown a positive correlation between sensory differences and internalizing problems. In this study, we hypothesized that sensory differences and suffering due to sensory differences mediates the relationships between autistic traits and internalizing problems.

**Methods:**

A total of 346 female Japanese university students completed questionnaires regarding their autistic traits, suffering due to sensory differences, and internalizing problems. Moreover, 114 participants completed a questionnaire related to sensory differences.

**Results:**

Autistic traits were correlated with Low Registration and Sensation Avoiding. These sensory differences were also correlated with suffering due to sensory differences and internalizing problems. Moreover, path analysis indicated that the higher the suffering due to Low Registration and Sensation Avoiding was, the greater the internalizing problems in those who showed these sensory differences.

**Conclusions:**

Female university students with serious suffering due to sensory differences may need support in managing their suffering and internalizing problems. Further research will help suggest support that these people require, at school and elsewhere.

**Supplementary Information:**

The online version contains supplementary material available at 10.1186/s40359-022-00854-0.

## Background

The term “autism spectrum disorder” (ASD) was introduced in the fifth edition of the Diagnostic and Statistical Manual of Mental Disorders (DSM-5). ASD is diagnosed by persistent deficits in social communication and interaction across multiple contexts, and restricted or repetitive patterns of behavior [1]. To measure the degree to which an adult with normal intelligence has these traits, Baron-Cohen and colleagues [2] developed the autism spectrum quotient (AQ). Intensity of autistic features vary among autistic people; autistic traits are distributed as a continuum in the general population [3, 4]. Furthermore, autistic traits exhibit stability from childhood through early adulthood in individuals with and without ASD [5].

The risk of secondary problems may be elevated if a person with strong autistic traits cannot have access to appropriate support. In fact, it has been reported that individuals with ASD exhibit greater internalization of problems, including depression and anxiety, than those without ASD, and they often develop co-occurring disorders such as anxiety disorder and mood disorders [6, 7]. Moreover, even in the general population, the stronger an individual’s autistic traits were, the greater the internalization of problems. For example, there is a relationship between autistic traits and internalizing problems in general elementary school students [4, 8]. University students also exhibit a correlation between higher anxiety and more severe autistic traits [9]. Because individuals with strong autistic traits but no diagnosis of ASD are at high risk of these psychiatric issues, further study on the general population to provide appropriate support is needed [4]. However, few studies have investigated students in general. Therefore, more detailed research on the relationship between autistic traits and internalizing problems is required.

Particularly in regard to females, studies and support may be needed for individuals who are not diagnosed with ASD but have high autistic traits because there is evidence of a diagnostic bias that girls who meet the criteria for ASD are at risk of not receiving a clinical diagnosis [10]. One reason females are underdiagnosed is masking. For example, it has been reported that in females with ASD, milder repetitive stereotyped behaviors and less severe difficulties at school may lead to under-recognition of ASD [11]. However, females with ASD need support in regard to different aspects because they often show other problems, such as greater internalization of problems and more sensory differences than males with ASD [12]. Therefore, although many studies have focused on males, further studies on females are needed to provide the necessary support for females with ASD as well as those with strong autistic traits.

Among autistic traits, sensory differences have been attracting attention in recent years and were added as a diagnostic criterion of ASD in the DSM-5. They are described as “hyper- or hypo-reactivity to sensory input or unusual interest in sensory aspect of the environment” [1]. Indeed, many empirical studies have found that individuals with ASD have sensory differences [13–15]. It has been reported that there is no differentiation by age [15]. Regarding the general population, it has been reported that people who have not been diagnosed with ASD but have high levels of autistic traits have sensory differences [16, 17]. Therefore, even in the general population, individuals with high autistic traits may also have sensory differences. If autistic traits are related to sensory differences in the general population, support related to sensory differences should also be provided to individuals with high autistic traits. However, only a small number of studies have focused on sensory differences in the general population, and related support is mainly provided to individuals with ASD. Hence, more detailed research is required regarding sensory differences in this population.

One of the most widely used tools to assess sensory differences is the Sensory Profile (SP) questionnaire based on Dunn’s model [18]. Dunn proposed that there is an interaction between neuroscience and behavioral concepts. The SP has four quadrants: Low Registration, Sensation Seeking, Sensory Sensitivity, and Sensation Avoiding. Low Registration is a combination of high neurological thresholds and passive behavioral responses while Sensation Seeking is a combination of high neurological thresholds and active behavioral responses. In the case of the other two quadrants, Sensory Sensitivity is a combination of low neurological thresholds and passive behavioral responses while Sensation Avoiding is a combination of low neurological thresholds and active behavioral responses. Some studies using the SP series have suggested that many individuals with ASD demonstrate a variety of sensory patterns and relatively more Low Registration and Sensation Avoiding behaviors [14, 19].

Sensory differences could also be associated with internalizing problems. It was indicated that the higher a person’s sensory differences, the higher the anxiety they experienced, even though most were not diagnosed with ASD [20]. Moreover, there was a significant correlation between sensory differences and depressive symptoms among university students [21]. Kasahara [22] pointed out that problems related to sensory differences are stressful because the distress is not understood by others. Robertson and Simmons [23] claimed that if someone feels negative emotions toward others as a result of uncomfortable sensory experiences, that person may suffer increased anxiety. However, the mechanism of the relationship between sensory differences and internalizing problems has not been clarified. Therefore, it is necessary to investigate this relationship in more detail. Furthermore, sensory differences may be particularly distressing in females, because it has been indicated that sensory differences are higher in females than in males, both for individuals with ASD [12] and without [20]. For this reason, it is necessary to further investigate this issue in females.

Regarding the relationship between sensory differences and internalizing problems, it may be necessary to focus not only on the frequency of responses to sensory experiences that are measured by the SP series but also on the subjective suffering resulting from different responses to daily sensory experiences. Internalization of problems seems to occur when a person possesses autistic traits and also experiences suffering resulting from ASD because this suffering can be associated with depression and anxiety [24, 25]. Similarly, internalization of problems may occur when a person responds atypically to sensory experiences and thus encounters suffering. Many people with strong autistic traits in the general population reported that they had difficulty with sensory stimuli [23]. It was also reported that sensory differences affect autistic students’ learning, such as disrupting their concentration [26], and autistic children’s distress while at school has been shown to be due to sensory symptoms [27]. Thus, suffering due to sensory differences is common in people with ASD and in the general population. This suffering may cause internalizing problems: research has shown that in regard to adolescents with ASD, when students suffer from sensory differences, they feel anxious and uncomfortable [26]. Therefore, suffering due to sensory differences may play an important role in clarifying the relationship between sensory differences and internalizing problems.

Furthermore, if suffering adversely affects the lives of people with sensory differences, support based on suffering due to sensory differences could be useful. In particular, students may need directed support for their suffering because of sensory differences at school [26]. Robertson and Simmons [23] also suggested that adapting educational environments to reduce sensory stressors would benefit everyone and increase inclusiveness because people in the general population, in addition to people with ASD, have suffered due to sensory differences. Therefore, it is necessary to investigate the frequency of such suffering in students to understand when and where support is needed at school.

In the present study, we developed and administered a scale to assess the frequency of suffering due to sensory differences in daily experiences, especially in university life for students. We studied university students to investigate their subjective suffering at school due to sensory differences using self-report measures. Furthermore, we tested whether there is a higher internalization of problems with (1) the higher the autistic traits, (2) the higher the sensory differences, and also (3) the suffering due to sensory differences in Japanese female university students. To investigate the last aspect, we created a hypothetical model that autistic traits affect sensory differences and sensory differences affects the internalization of problems indirectly through an suffering due to sensory differences.

## Methods

### Participants

This investigation was included in a health survey conducted at Ochanomizu University, a women’s university in Japan. A series of paper-and-pencil questionnaires was distributed to approximately 2000 female university students during their classes, and 346, aged 18–47 (mean age = 20.75, *SD* = 2.54), completed them; the questionnaires could be completed at any time and were submitted to collection boxes. Subsequently, 114 of these participants (age 18–37, mean = 20.19, *SD* = 1.90) also completed another paper-and-pencil series, including the Adult/Adolescent Sensory Profile (AASP), during other classes. We conducted analyses of these participants related to the AASP. Neither series of questionnaires included any questions regarding diagnosis of ASD or other developmental disorders. Participation in the survey was voluntary. Each participant provided written informed consent prior to survey participation. This study was approved by the ethics committee of Ochanomizu University and conducted in accordance with the Declaration of Helsinki.

### Measures

1. Autistic traits: We used the Japanese version of the short autism spectrum quotient (AQ-10) to measure autistic traits. Allison and colleagues [28] selected 10 items from the original AQ with the greatest discriminatory power and developed the AQ-10. The AQ-10 has adequate validity to measure autistic traits within the general population [29]. We extract these 10 items from the Japanese version of AQ [30]. It is then scored on a four-point Likert scale and is converted to a binary format (1 = definitely agree, 2 = slightly agree, 3 = slightly disagree, 4 = definitely disagree). Higher scores indicated stronger autistic traits, and the total score of the 10 items was used to indicate the degree of autistic traits. The Cronbach’s alpha was 0.69.
2. Sensory differences: AASP is a 60-item self-report questionnaire measuring responses to sensory experiences in daily life [31]. Hagiwara and colleagues [32] developed the Japanese version of AASP and demonstrated its reliability and validity to be adequate. Participants were instructed to indicate the frequency of their responses to sensory experiences on a five-point Likert scale (1 = almost never, 2 = seldom, 3 = occasionally, 4 = frequently, 5 = almost always). Higher scores indicated greater sensory differences. The scores were grouped into four quadrants, each of which included 15 items: Low Registration, Sensation Seeking, Sensory Sensitivity, and Sensation Avoiding. The Cronbach’s alphas in the current study were 0.74 for Low Registration, 0.61 for Sensation Seeking, 0.75 for Sensory Sensitivity, and 0.77 for Sensation Avoiding.
3. Suffering due to sensory differences in university: We developed seven items in order to measure the suffering in university life resulting from sensory differences described in the AASP. For example, the statement “I cannot concentrate on the unsteady or fast moving visual images I see in class” was developed from “I am bothered by unsteady or fast moving visual images in movies or TV” included in Sensory Sensitivity. Responses were scored on a four-point Likert scale (0 = not applicable/I do not suffer, 1 = I suffer a little, 2 = I suffer, 3 = I suffer a great deal). Although the AASP measures the response frequency to sensory experiences, the scale we developed was intended to assess the subjective suffering that a person experiences (i.e., the extent to which they perceive suffering from a sensory experience). All items were developed from the items included in the Low Registration (2 items), Sensory Sensitivity (3 items), and Sensation Avoiding (2 items) subscales of the AASP. Sensation Seeking in the AASP is constructed by items that seem not to cause suffering, such as ‘I like to wear colorful clothing,’ and include few items that could cause suffering in university life. Therefore, we did not include these items. Higher scores indicate greater suffering due to sensory differences in university. The questionnaire and its descriptive statistics can be found in Additional File 1.
4. Internalizing problems: The Japanese version of the Strength and Difficulties Questionnaire (SDQ) for those aged 18 and over is a 25-item self-report questionnaire [33, 34]. Each of five subscales includes five items. We used 2 subscales, emotional symptoms and peer problems to measure the internalization of problems. In this study, the total score of the 10 items was used as the score indicating the intensity of internalizing problems. These subscales are often used to measure internalizing problems, and there is theoretical and preliminary empirical support for combining emotional symptoms and peer problems into an internalizing subscale, especially in low-risk samples [35]. This combined subscale has higher validity and reliability than the emotional symptoms and peer problems subscales [35]. It is scored on a three-point Likert scale (0 = not true, 1 = somewhat true, 2 = certainly true), with higher scores indicating a greater internalization of problems. The Cronbach’s alpha in the current study was 0.68.

### Statistical analysis

We explored the factor structure of the questionnaire on suffering due to sensory differences at university using confirmatory factor analysis (CFA) and examined the internal consistency of the scale. Furthermore, the frequency and proportion of each statement regarding suffering due to sensory differences were calculated.

Correlation coefficients were calculated among autistic traits, sensory differences, suffering due to sensory differences, and internalizing problems. We used the false discovery rate (FDR) correction for multiple correlation testing [36]. Path analysis was used to estimate direct and indirect paths between these variables. We also tested the mediating effects of suffering due to sensory symptoms using 10,000 bootstrap samples in path analysis [37].

The levels of fit in the CFA and path analysis were assessed using several indices. Although non-significant χ^2^ values provide some evidence of acceptable model fit, the chi-square index is inadequate as a stand-alone fit index [38]. Therefore, we also examined several other indices. The goodness-of-fit index (GFI), adjusted goodness-of-fit index (AGFI), and comparative fit index (CFI) are typically used to indicate a good fit of the model to the data, with values around 0.95 or higher [39]. Root mean square error of approximation (RMSEA) values lower than 0.08 indicate an acceptable fit [40].

Data analysis was performed using SPSS 25.0 and Amos 25.0 (IBM Corp., Armonk, New York). The dataset for this analysis is available in the Additional File 3.

## Results

**Fig. 1:**
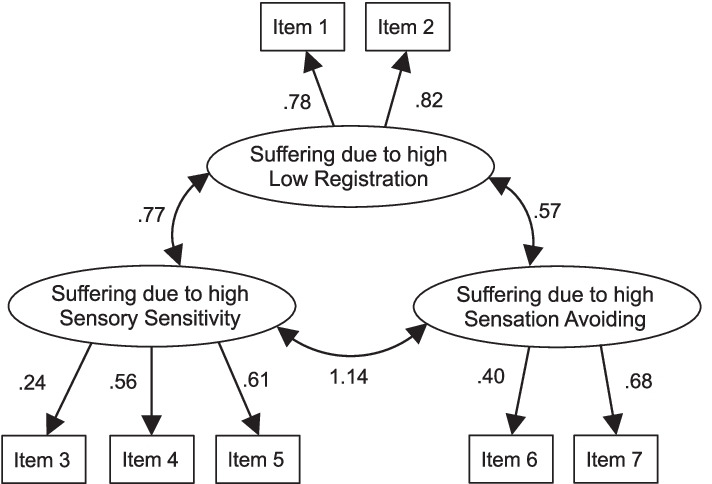
The results of CFA of suffering due to sensory differences. The model provided an acceptable fit to the data (χ.^2^ (11) = 28.20, *p* = .00, GFI = .98, AGFI = .95, CFI = .97, RMSEA = .07)

First, we performed CFA to examine the factor structure of the seven items related to suffering due to sensory differences at university. Because this scale was developed from the items included in the Low Registration, Sensory Sensitivity, and Sensation Avoiding subscales of the AASP, we predicted that the fit of a model that assumes three factors related to each quadrant would be a good fit. Each latent variable consists of two or three indicators. Five students with missing data were excluded from the CFA, and leaving 341 students ultimately included in this analysis. The results of CFA are shown in Fig. 1. Although the model provided a significant χ^2^ value (χ^2^ (11) = 28.20, *p* = 0.00), other indices indicated an acceptable fit for the data (GFI = 0.98, AGFI = 0.95, CFI = 0.97, RMSEA = 0.07). The Cronbach’s alphas were 0.78 for suffering due to Low Registration, 0.44 for Sensory Sensitivity, and 0.41 for Sensation Avoiding. Therefore, the total scores for the items of each factor were used in the analysis as a score indicating suffering due to each sensory differences quadrant.

The frequency and proportion of each statement regarding suffering due to sensory differences among students (*n* = 346) were calculated. As a result, the degree of suffering for some items was high, while for others, it was low (the percentages of students who selected from “I suffer a little” to “I suffer a great deal” for each item ranged from 24.6 to 49.4%). For example, “It is distressing to be in a crowded classroom or school cafeteria because I do not like to get too close to others” had a relatively high degree of difficulty; 26.88% of the participants selected “I suffer a little,” 13.87% selected “I suffer,” and 8.09% selected “I suffer a great deal.” For the statement “Sometimes I can’t catch what the teacher says during the class,” 32.08% of the participants selected “I suffer a little,” 11.40% selected “I suffer,” and 3.51% selected “I suffer a great deal.” In other words, about 50% of the participants selected responses ranging from “I suffer a little” to “I suffer a great deal” for these items. On the other hand, for the statement “I am distracted if there is a lot of noise around, such as in a crowded school cafeteria or in a large classroom before class,” 73.70% of the participants selected “not applicable” or “I do not suffer,” and few students reported suffering.

Next, the correlation coefficients were calculated between autistic traits, the four quadrants in the AASP, and internalizing problems (Table 1). The following analyses included 114 students who completed all of the questionnaires, including the AASP. Low Registration, Sensory Sensitivity, and Sensation Avoiding were significantly correlated with each other and with internalizing problems. However, Sensation Seeking did not significantly correlate with Sensory Sensitivity, Sensation Avoiding, or internalizing problems. Autistic traits were only significantly correlated with Low Registration among the four quadrants of the AASP. They also showed a trend toward correlation with Sensation Avoiding. In contrast, Sensation Seeking and Sensory Sensitivity did not correlate with autistic traits. These sensory differences were not included in the following analyses because this study aimed to investigate the relationship between internalizing problems and sensory differences related to autistic traits.

**Table 1:** Correlations between autistic traits, sensory differences and internalizing problems

			Sensory differences							
	1		2		3		4		5	
	*r*	*p*	*r*	*p*	*r*	*p*	*r*	*p*	*r*	*p*
1. Autistic traits										
*Sensory differences*										
2. Low registration	0.25	0.015								
3. Sensory seeking	–0.13	0.215	0.23	0.022						
4. Sensory sensitivity	0.16	0.135	0.47	< 0.001	0.07	0.514				
5. Sensation avoiding	0.19	0.070	.40	< 0.001	–0.05	0.657	0.72	< 0.001		
6. Internalizing problems	0.23	0.022	.36	< 0.001	–0.02	0.797	0.46	< 0.001	0.39	< 0.001

*n* = 114; *p*-values corrected for false discovery rate

Therefore, the total score for Low Registration and Sensation Avoiding was employed as an index, indicating sensory differences related to ASD. This index was significantly correlated with autistic traits and internalizing problems, and its internal consistency was sufficiently high (Cronbach’s alpha = 0.82). These sensory differences were presumed to cause suffering due to Low Registration and Sensation Avoiding. Hence, the total score for suffering due to Low Registration and Sensation Avoiding was employed as an index indicating suffering due to sensory differences related to ASD. This index had a Cronbach’s alpha of 0.64 and was significantly correlated with autistic traits and internalizing problems. The correlations among the variables are presented in Table 2.

**Table 2:** Correlations between age, autistic traits, sensory differences, suffering due to sensory differences, and internalizing problems

				1		2		3		4	
	*M*	*SD*		*r*	*p*	*r*	*p*	*r*	*p*	*r*	*p*
Age	20.19	1.90									
Autistic traits (10–40)	24.17	3.85	FDR adjusted	− 0.06	0.602						
			CMV adjusted								
Sensory differences related to ASD (30–150)	68.35	13.09	FDR adjusted	− 0.18	0.101	0.26	0.015				
			CMV adjusted			0.20	0.035				
Suffering due to sensory differences related to ASD (0–21)	2.42	2.22	FDR adjusted	0.01	0.882	0.22	0.033	0.50	< 0.001		
			CMV adjusted			0.16	0.098	0.46	< 0.001		
Internalizing problems (0–20)	11.74	3.55	FDR adjusted	− 0.13	0.220	0.23	0.025	0.44	< 0.001	0.37	< 0.001
			CMV adjusted			0.17	0.066	0.40	< 0.001	0.32	< 0.001

*n* = 114

Because this study only used self-report measures, these relationships are vulnerable to correlation inflation due to common method variance (CMV). Therefore, we used the marker variable technique to control for the effects of CMV [41]. Lindell and Whitney [41] proposed using the following equations to calculate a CMV-adjusted correlation, *r*_*Yi.M*_, and its *t*-statistic:

$$r_{Yi.M} = \left( {r_{Yi} {-}r_{s} } \right)/\left( {{1 }{-}r_{s} } \right)$$

$$t{\text{statistic}} = \, r_{Yi.M} / \, \surd \left[ {\left( {{1 }{-}r^{2}_{Yi.M} } \right)/\left( {n{-}{ 3}} \right)} \right]$$

*r*_*Yi*_ is the observed correlation suspected of being contaminated by CMV, and *r*_*s*_ is the smallest observed correlation between the substantive variables and the marker variable that is chosen a priori and is expected to be theoretically unrelated to at least one substantive variable. However, if an a priori marker variable is not included, investigators can use the smallest positive correlation in the dataset as an estimate of *r*_*s*_.

In this study, we used the smallest positive correlation as a marker variable for all of the variables used in Tables 1 and 2, excepting age. The CMV-adjusted correlation variables and *p*-values are presented in Table 2. After adjusting for CMV, correlations between autistic traits and sensory differences and among sensory differences, suffering, and internalizing problems remained significant. However, correlations between autistic traits and suffering and between autistic traits and internalizing problems were not significant *n* = 144.

**Fig. 2:**
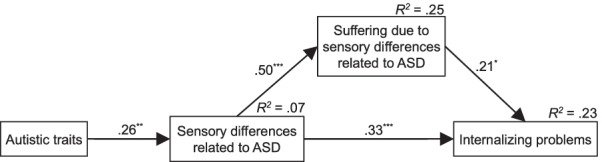
Structural model of autistic traits, sensory differences, suffering due to sensory differences, and internalizing problems. The model provided a good fit to the data (χ^2^ (2) = 2.57, *p* = .28, GFI = .99, AGFI = .94, CFI = .99, RMSEA = .05). ^*^p < .05, ^**^p < .01, ^***^p < .001

Path analysis was then used to estimate the direct and indirect paths between autistic traits, sensory differences, suffering due to sensory differences related to ASD, and internalizing problems. In the path analysis, 105 participants with no missing values were included in the analysis. The score of the sensory differences related to ASD was used as the score indicating the sensory differences, and the score of suffering due to sensory differences related to ASD were used as a score indicating suffering due to sensory differences. The results of the path analysis are shown in Fig. 2. The path analysis model provided a good fit to the data (χ^2^ (2) = 2.57, *p* = 0.28, GFI = 0.99, AGFI = 0.94, CFI = 0.99, RMSEA = 0.05). This result indicates that the greater the suffering due to sensory differences is, the greater the internalization of problems in those who score higher on sensory differences related to ASD. We further examined whether suffering due to sensory differences mediated the effect of sensory differences on internalizing problems. The indirect effect (0.11) was smaller than the direct effect (0.33). However, the 95% confidence interval of the indirect effect based on 10,000 bootstrap samples ranged from 0.02 to 0.25, suggesting that the indirect effect was significant.

## Discussion

In this study, we developed a scale to measure suffering due to sensory differences and investigated the relationship among autistic traits, sensory differences, suffering due to sensory differences, and internalizing problems. Only Low Registration and Sensation Avoiding were correlated with autistic traits, and they also correlated with suffering due to sensory differences and internalizing problems. Path analysis revealed that suffering due to sensory differences related to ASD significantly mediated the relationship between sensory differences related to ASD and internalizing problems.

Some studies have reported that many individuals with ASD showed particularly Low Registration and Sensation Avoiding [14, 19]. This study also indicated that autistic traits were significantly positively correlated with the total score of Low Registration and Sensation Avoiding in healthy individuals. Since some studies have indicated that sensory differences are related to other developmental disorders and other mental disorders [42–44], it is also necessary to investigate the relationship between sensory differences and conditions other than ASD.

There was a significant positive correlation between autistic traits and internalizing problems, which is consistent with previous studies on the general population [4, 8]. This result supported the hypothesis that the higher the indication of autistic traits, the greater the internalization of problems.

Regarding the relationship between sensory differences and internalizing problems, previous studies using the AASP showed that total score was associated with anxiety [20], and that there were correlations between the total score of Sensory Sensitivity and Sensation Avoiding, and anxiety, as well as between Low Registration and symptoms of depression in adolescents [45]. We also found significant positive correlations between internalizing problems and Low Registration, Sensory Sensitivity, and Sensation Avoiding. Our result coincides with the study of Pfeiffer and colleagues [45], where Sensation Seeking was not highly correlated with internalizing problems. Because in the AASP, Sensation Seeking included few items that asked about experiences that are likely to cause suffering in daily life, that our study found no correlation between Sensation Seeking and internalizing problems was expected. Hence, our hypothesis (i.e., the higher the sensory differences, the higher the internalization of problems) was supported for Low Registration, Sensory Sensitivity, and Sensation Avoiding.

The results of path analysis indicated that the higher the suffering due to sensory differences, the greater the internalization of problems is in those who scored high on Low Registration and Sensation Avoiding. Previous studies have indicated that sensory differences correlate with internalizing problems in the general population [20, 21] but have not revealed the relationship between suffering due to sensory differences and these variables. However, this study indicates that suffering due to sensory differences significantly mediates the relationship between sensory differences and internalizing problems. As the hypothesis was supported, the higher the suffering due to sensory differences is, the greater the internalization of problems, consisting of emotional symptoms and peer problems. Therefore, suffering due to sensory differences is related to anxiety and depression. Moreover, students who experience suffering may have difficulty building a relationship with their peers at university because they have difficulty following the classes. As previously mentioned, suffering due to sensory differences can create obstacles when taking classes, causing stress at university. However, the teaching staff and fellow students at the university may not notice this suffering in class participants. At present, there is little individual support and class styles are diverse. Therefore, it is necessary to determine and provide adequate support for such students.

In this study, we have shown that university students can need support when they have suffering due to sensory differences. Because there were significant positive correlations between internalizing problems and Low Registration, Sensory Sensitivity, and Sensation Avoiding in this study, supports can be needed for suffering related especially to these quadrants. Regarding Sensory Sensitivity, there was no significant correlation with autistic traits, but it was significantly correlated with internalizing problems. Therefore, regardless of the degree of autistic traits, all female university students with high Sensory Sensitivity score may need support. For people with a high Sensory Sensitivity score, concentration can be sustained by providing support strategies such as a reduction in sensory stimulation, incorporating active content to aid concentration, making plans beforehand, and taking a rest [31]. Therefore, students may improve their concentration in class by reducing unnecessary sensory stimulation while increasing speaking opportunities and group discussion.

The total score for Low Registration and Sensation Avoiding significantly correlated with autistic traits. In addition, the greater the suffering due to sensory differences was, the greater the internalization of problems in those who showed these sensory differences. According to Brown and Dunn [31], for people with a high score for Low Registration, it is useful support to use visual and auditory cues to sharpen and increase the intensity of important stimuli, and to slow their presentation speed. On the other hand, for people with a high Sensation Avoiding score, it is useful support to reduce unnecessary sensory stimulation, to take a rest, to avoid crowds, find a quiet place, and maintain consistency [31]. Therefore, especially for female university students with strong autistic traits, it would be useful to offer small classes, emphasize important stimuli, and maintain a predictable and consistent environment. If it is difficult to change the overall form of the class, individual support according to their suffering maybe useful. For example, to allow them to record the class or attend online. Hence, it is important for faculty members to devise ways to proceed with classes and provide individual support to female university students, especially those with strong autistic traits. These measures may reduce the suffering due to sensory differences in university and partially mitigate internalizing problems.

Furthermore, the present study suggests that suffering due to sensory differences positively correlate with internalizing problems. Therefore, university students with serious suffering due to sensory differences may need support for managing their suffering and internalizing problems elicited by them. Above all, it may be necessary to provide support where responses to items in the questionnaire revealed a relatively high degree of suffering. For example, positive responses to “Sometimes I cannot catch what the teacher says during the class” and “It is distressing to be in a crowded classroom or school cafeteria because I do not like to get too close to others” are useful warnings. On the other hand, the need for support for female university students may be low for items where students report little suffering. However, the assessment of suffering due to sensory differences are based on the AASP, which uses items related to sensory abnormalities that are common in ASD individuals but rarely seen in general population. Therefore, further research on females with ASD is needed because ASD students may suffer even for items where many students reported little or no suffering in this study.

The current study has four limitations to note. First, the sample was limited. Although it is significant that we investigated female university students in the general population for this study, it is necessary to conduct an investigation of male university students and determine if there are gender differences. In addition, it is also necessary to study the general population across a wider range of age groups. Second, there were few items in the questionnaire relating to suffering due to sensory differences at university. We will be able to determine more methods for supporting students by developing more items and analyzing their responses. Furthermore, although we developed the suffering scale based on the AASP and did not include the items in the Sensation Seeking subscale, some items in Sensation Seeking, such as “Seeks all kinds of movement and this interferes with daily routines (for example, can’t sit still, fidgets)” in the original SP, may indicate sensory experiences that induce suffering. Further studies including items of Sensation Seeking and in more varied situations are needed. We also need to standardize this questionnaire in relation to suffering due to sensory differences because we could not sufficiently confirm its reliability and validity in this study. Third, because this study was cross-sectional, it is necessary to conduct a longitudinal study to discover and investigate the causal relationships between each variable. Fourth, the Cronbach’s alphas of the AQ-10, some subscales of suffering due to sensory differences, and internalizing problems measured by the SDQ in this study were not sufficiently high. A previous study reported that the Cronbach’s alpha for AQ-10 was 0.72 in the clinical group and 0.45 in the control groups [46]. The Cronbach’s alpha in our study was 0.69, which may be because our study was conducted with healthy individuals. Further research using more reliable measures, such as the AQ-50, are necessary. Regarding suffering, all subscales included two or three items. More items related to suffering due to sensory differences need to be developed. Moreover, further research should be conducted with individuals who have higher sensory differences because few participants suffered from some items included in the suffering scale. Regarding SDQ, the validation study for the internalizing subscale on the SDQ reported that the Cronbach’s alpha of internalizing problems was 0.66 in the self-reported SDQ and that it indicates better internal reliability than the subscales covering emotional symptoms and peer problems [35]. The Cronbach’s alpha of internalizing problems in our study was 0.68, indicating some degree of reliability. Moreover, further research using more detailed scales is needed to investigate how suffering due to sensory differences relates to anxiety, depression, and peer problems because the SDQ measures relatively broad internalizing problems.

Further research of the general population could reveal sensory differences for factors other than autistic traits, and the resultant suffering. Additionally, this could help design support systems for people with high sensory differences in school and elsewhere, enabling them to lead a better life.

## Conclusions

This study for the first time showed that suffering due to sensory differences mediate the relationship between sensory differences and internalizing problems. Autistic traits were correlated with Low Registration and Sensation Avoiding, and the higher the suffering due to sensory differences was, the greater the internalization of problems in those who scored high on these indicators. Suffering from sensory differences can form obstacles in classes, which induces stress at the university. Therefore, it is necessary to develop and provide adequate support to students who suffer from sensory differences.

## Supplementary Information

**Additional file 1**: The items of the suffering due to sensory differences in university, and mean and SD of each item.

**Additional file 2**: The questionnaire of the suffering due to sensory differences in university.

**Additional file 3**. Raw data.

## Data Availability

The dataset for this analysis is available in the Additional file 3.

## Abbreviations

AASP: Adult/adolescent sensory profile
AGFI: Adjusted goodness-of-fit index
AQ: Autism spectrum quotient
AQ-10: Short autism spectrum quotient
ASD: Autism spectrum disorder
DSM-5: 5Th edition of the diagnostic and statistical manual of mental disorders
CFA: Confirmatory factor analysis
CFI: Comparative fit index
CMV: Common method variance
FDR: False discovery rate
GFI: Goodness-of-fit index
RMSEA: Root mean square error of approximation
SDQ: Strength and suffering questionnaire
SP: Sensory profile
