# Supplementary material for: Mediating role of sensory differences in the relationship between autistic traits and internalizing problems
Source: BMC Psychol. 2022 Jun 13;10:148. doi: 10.1186/s40359-022-00854-0 (PMC9190171; doi:10.1186/s40359-022-00854-0)
Supplement: Supplementary file 1 — Additional file 1: The items of the suffering due to sensory differences in university, and mean and SD of each item. [file 40359_2022_854_MOESM1_ESM.docx]

**Additional file 1.** The items of the suffering due to sensory differences in university, and mean and SD of each item.

| Items | Mean | SD |
| --- | --- | --- |
| Suffering due to high Low Registration |  |  |
| 1. I find it difficult to follow the classes because I am slow to follow the steps of activities and work.  活動や作業の手順を追うのが遅いせいで、授業についていけないことがある | 0.53 | 0.76 |
| 2. Sometimes I cannot catch what the teacher says during the class.  授業中に先生の言っていることを聞き取れないことがある | 0.69 | 0.78 |
| Suffering due to high Sensory Sensitivity |  |  |
| 3. There are few foods I can buy because I dislike the tastes and textures of much of the food and drink sold at the school cafeteria.  学食や購買で売っている食べ物や飲み物は、味や食感が嫌いなものが多いので、買えるものが少ない | 0.38 | 0.71 |
| 4. I cannot concentrate on the unsteady or fast moving visual images I see in class.  授業で見る映像が不安定だったり、画像が速かったりすると内容が頭に入ってこない | 0.41 | 0.69 |
| 5. I am distracted if there is a lot of noise around, such as in a crowded school cafeteria or in a large classroom before class.  お昼休み中の混んでいる食堂や授業前の大教室の中など、まわりが騒々しいと混乱してしまう | 0.35 | 0.69 |
| Suffering due to high Sensation Avoiding |  |  |
| 6. I do not want to enter a classroom with a strong smell, such as other people’s perfume.  他の人の香水などの強いにおいがする教室に入りたくない | 0.48 | 0.77 |
| 7. It is distressing to be in a crowded classroom or school cafeteria because I do not like to get too close to others.  他の人がすぐそばにいるのが嫌なので、混み合った教室や食堂にいるのが不快だ | 0.72 | 0.94 |
