# Supplementary material for: Mediating role of sensory differences in the relationship between autistic traits and internalizing problems
Source: BMC Psychol. 2022 Jun 13;10:148. doi: 10.1186/s40359-022-00854-0 (PMC9190171; doi:10.1186/s40359-022-00854-0)
Supplement: Supplementary file 2 — Additional file 2: The questionnaire of the suffering due to sensory differences in university. [file 40359_2022_854_MOESM2_ESM.docx]

**Additional file 2.** The questionnaire of the suffering due to sensory differences in university.

大学生活を送る上で、それぞれの項目の内容についてどの程度困っているかを判断し、最もあてはまる数字に〇をつけてください。

**2**

困っている

**3**

とても困っている

**1**

少し困っている

**0**

該当しない/困っていない

| 1. 活動や作業の手順を追うのが遅いせいで、授業についていけないことがある | 0 | 1 | 2 | 3 |
| --- | --- | --- | --- | --- |
| 1. 授業中に先生の言っていることを聞き取れないことがある | 0 | 1 | 2 | 3 |
| 1. 学食や購買で売っている食べ物や飲み物は、味や食感が嫌いなものが多いので、買えるものが少ない | 0 | 1 | 2 | 3 |
| 1. 授業で見る映像が不安定だったり、画像が速かったりすると内容が頭に入ってこない | 0 | 1 | 2 | 3 |
| 1. お昼休み中の混んでいる食堂や授業前の大教室の中など、まわりが騒々しいと混乱してしまう | 0 | 1 | 2 | 3 |
| 1. 他の人の香水などの強いにおいがする教室に入りたくない | 0 | 1 | 2 | 3 |
| 1. 他の人がすぐそばにいるのが嫌なので、混み合った教室や食堂にいるのが不快だ | 0 | 1 | 2 | 3 |

(English version)

Please circle the number in the column that best represents your suffering in the university life.

**3**

I suffer a great deal

**0**

Not applicable/I do not suffer

**1**

I suffer a little

**2**

I suffer

| 1. I find it difficult to follow the classes because I am slow to follow the steps of activities and work. | 0 | 1 | 2 | 3 |
| --- | --- | --- | --- | --- |
| 1. Sometimes I cannot catch what the teacher says during the class. | 0 | 1 | 2 | 3 |
| 1. There are few foods I can buy because I dislike the tastes and textures of much of the food and drink sold at the school cafeteria. | 0 | 1 | 2 | 3 |
| 1. I cannot concentrate on the unsteady or fast moving visual images I see in class. | 0 | 1 | 2 | 3 |
| 1. I am distracted if there is a lot of noise around, such as in a crowded school cafeteria or in a large classroom before class. | 0 | 1 | 2 | 3 |
| 1. I do not want to enter a classroom with a strong smell, such as other people’s perfume. | 0 | 1 | 2 | 3 |
| 1. It is distressing to be in a crowded classroom or school cafeteria because I do not like to get too close to others. | 0 | 1 | 2 | 3 |
